# A curated dataset of Abstract Syntax Tree-based structural dependency graphs and object-oriented metrics for 21 open-source Java systems

**DOI:** 10.1016/j.dib.2026.113290

**Published:** 2026-09-24

**Authors:** Marcos Cordeiro de Brito, Leandro Augusto da Silva

**Affiliations:** Mackenzie Presbyterian University, R. da Consolação, 930 - Consolação, São Paulo - SP, 01302-907, São Paulo, Brazil

**Keywords:** Static analysis, Software networks, Software modularity, Architectural recovery, Reproducible benchmarks, Community detection

## Abstract

This article describes a curated dataset of structural dependency graphs and object-oriented metrics extracted from 21 open-source Java systems. Each repository was analyzed at a fixed commit using a deterministic AST-based static analysis pipeline. For every project, the dataset provides a weighted directed GraphML file encoding class-to-class structural dependencies, with node-level metrics (LCOM4, CBO, RFC, NOM, NOA) embedded as node attributes, together with execution metadata documenting repository URL, commit hash, extraction date, tool and dependency versions, and a configuration hash that makes the analysis parameters independently verifiable. The selected systems cover distinct domains, including build tools, frameworks, distributed platforms, and utility libraries, and range in size from 43 to 29,695 classes. All artifacts correspond to dataset version v1.0.1 and are publicly available through a Zenodo repository (DOI: https://doi.org/10.5281/zenodo.18744312). The dataset was generated using JASTG v1.0.0 (commit b62a943), providing version-level traceability between code and data. The dataset is intended for use in graph-based modularity analysis, community detection, and architectural structure mining on Java code representations.

Specifications Table

**Table:** 

Subject	Computer Sciences
Specific subject area	Structural dependency graphs and object-oriented metrics for Java systems
Type of data	Graph (GraphML), Table (CSV), JSON metadata Raw, Processed
Data collection	Java repositories were cloned at fixed commits and parsed using a deterministic AST-based static analysis pipeline (JASTG v1.0.0, commit b62a943). Explicit typed dependencies between classes were extracted and represented as weighted directed graphs. Node-level object-oriented metrics were computed and embedded as node attributes, and execution metadata was exported for each system.
Data source location	Public GitHub repositories; dataset hosted on Zenodo
Data accessibility	Repository name: Zenodo (dataset release version: v1.0.1) Data identification number: 10.5281/zenodo.18744312 (concept DOI, resolving to the latest version; version-specific doi:10.5281/zenodo.21221557) Direct URL to data: https://doi.org/10.5281/zenodo.18744312 Instructions for accessing these data: Publicly available without restriction and permanently archived under DOI versioning.
Related research article	Marcos Cordeiro de Brito Jr., Leandro Augusto da Silva. JASTG: Java AST Structural Graph — A tool for reproducible extraction of structural dependency graphs from Java systems. SoftwareX, 35:102,802, 2026. https://doi.org/10.1016/j.softx.2026.102802.

## 1 Value of the Data

• The dataset provides a ready-to-use collection of structural dependency graphs for 21 Java systems spanning heterogeneous domains and size scales, reducing the effort required by researchers to collect, parse, and standardize source code repositories for empirical studies in software engineering.
• The graphs can serve as a controlled benchmark for evaluating algorithms in community detection, modular decomposition, and architectural recovery, supporting researchers in software engineering, graph mining, and network analysis who seek standardized inputs for comparative studies.
• The co-located object-oriented metrics (LCOM4, CBO, RFC, NOM, NOA) allow researchers to investigate relationships between structural coupling, cohesion, and design quality without requiring additional tooling or reprocessing of the source repositories.
• Typical usage scenarios include: (i) benchmarking community detection algorithms — loading a GraphML file into NetworkX or igraph, applying algorithms such as Louvain or Leiden, and comparing modularity scores across systems of different scales; (ii) studying metric–modularity relationships — joining the node-level coupling and cohesion attributes (e.g., CBO, LCOM4) with community assignments to investigate how design quality relates to emergent modular structure; and (iii) comparative architectural recovery studies — evaluating clustering-based recovery techniques on systems spanning four orders of magnitude in size and heterogeneous application domains.
• Unlike corpora that distribute source code or bytecode and require users to build their own extraction pipelines (e.g., the Qualitas Corpus [1]), this dataset provides analysis-ready graph representations with metrics embedded in the same files, fixed commit hashes, and DOI-versioned immutable releases, enabling reproducible cross-study comparisons without any parsing infrastructure.
• Full provenance metadata, including fixed commit hashes, tool and dependency versions, extraction date, and a configuration hash, enables independent replication and dataset extension, inviting other researchers to contribute additional systems or produce comparable datasets using the same pipeline.

## 2 Background

This data article complements the description of the JASTG tool [2] by providing the dependency graph dataset and metrics generated through its application to 21 open-source Java systems. Structural representations of software systems have been widely adopted in software engineering research to investigate coupling, modularity, and architectural organization. Graph-based abstractions, where nodes represent software entities and edges represent dependencies, have been extensively used in architecture recovery and modularity analysis [[3], [4], [5]]. Object-oriented metrics are commonly used to characterize software design quality, including the Chidamber and Kemerer suite (CBO, RFC, LCOM) complemented by size measures such as NOM and NOA [6]. Graph-based community detection further supports the identification of cohesive modules in software networks [[7], [8], [9]]. Despite their widespread use, publicly available benchmarks that combine fixed repository commits, standardized graph representations, and associated metrics remain scarce, and existing datasets often lack versioning or traceability guarantees. The present dataset addresses this gap using JASTG [10], a deterministic AST-based pipeline aligned with established static analyzers such as Spoon and WALA [11,12], and is publicly archived on Zenodo [13].

## 3 Data Description

The dataset repository contains, for each of the 21 analyzed projects, a directory projects/<project>/ with two files:

• A GraphML file (graph_<project>.graphml) representing a weighted directed class dependency graph, with the node-level object-oriented metrics (LCOM4, CBO, RFC, NOM, NOA) embedded as node attributes and the full run provenance embedded as graph-level attributes, making each file self-contained.
• Execution metadata (metadata_<project>.json) including repository URL, commit hash, tool and dependency versions, execution environment, extraction date, a SHA-256 configuration hash, and parse statistics.

At the dataset level, the repository root contains projects.csv (the input manifest listing repository URLs, licenses, release tags, and commit hashes of the analyzed systems), benchmark_summary.csv (consolidated graph-level statistics for all 21 systems), data_dictionary.csv (a machine-readable schema describing every column and attribute of every file), and a README with loading examples for NetworkX, igraph, and Gephi.

In the GraphML files, nodes carry a label attribute of the form <project>/<fully.qualified.ClassName>, where nested classes follow the JVM $ convention (e.g., Outer$Inner), together with the five object-oriented metrics; edges carry a weight attribute encoding the number of distinct typed dependency occurrences. The following fragment, simplified for readability (attribute keys shown by name), reproduces real values from the jpetstore-6 graph:

**Table:** 

<node id="4">
<data key="label">jpetstore-6/org.mybatis.jpetstore.domain.Cart</data>
<data key="LCOM4">1</data>
<data key="CBO">2</data>
<data key="RFC">29</data>
<data key="NOM">10</data>
<data key="NOA">3</data>
</node>
<edge source="43″ target="42">
<data key="weight">8</data>
</edge>

The companion metadata file duplicates the provenance fields so that they can be inspected without loading the graph. The complete record for jpetstore-6 is:

**Table:** 

{
"project_url": "https://github.com/mybatis/jpetstore-6.git",
"jastg_version": "1.0.0",
"python_version": "3.14.3 (main, Feb 13 2026, ...)",
"platform": "Linux-6.18.9-arch1-2-x86_64-with-glibc2.43",
"javalang_version": "0.13.0",
"networkx_version": "3.6.1",
"config_hash": "40a80e9f4c257036e55a1a5e0343ec978ebaff44df5e807611408dd6fec93650",
"run_date": "2026-02-23T14:04:26.702394+00:00",
"commit_hash": "41207bd96c35dce3e746d4af0fdf343f4560ea68",
"num_classes": 43,
"num_edges": 98,
"total_java_files": 43,
"parse_errors": 0,
"directed": true,
"weighted": true
}

Table 1 lists the projects included in the dataset together with their licenses, analyzed commits, and basic graph sizes. Additional statistics such as density, average degree, and maximum degree are available in the CSV summary file hosted in the repository.

**Table 1:** Summary of analyzed projects (commit hashes truncated to 10 characters). Full commit hashes are available in projects.csv and benchmark_summary.csv in the Zenodo dataset repository (DOI: https://doi.org/10.5281/zenodo.18744312).

Project	License	Commit	#Classes	#Edges
ant	Apache-2.0	6a8f02c192	1770	5610
camel	Apache-2.0	069410104f	29,695	86,754
commons-collections	Apache-2.0	40c0cb45fd	924	2532
commons-io	Apache-2.0	c5c152e050	662	1417
commons-lang	Apache-2.0	b56c6882fb	960	1588
commons-math	Apache-2.0	39ca2ebfff	1279	5127
dropwizard	Apache-2.0	e43702c712	1143	2152
eclipse-collections	EPL-2.0	7070213d39	3169	14,804
flink	Apache-2.0	fdc2686e33	21,078	114,462
gson	Apache-2.0	a8e6c962ea	683	1545
javalin	Apache-2.0	9c3461f16e	74	69
jpetstore-6	Apache-2.0	41207bd96c	43	98
junit5	EPL-2.0	e92b405556	2347	3665
kafka	Apache-2.0	4d4938cf1f	6962	38,386
maven	Apache-2.0	71bd10f28c	3024	6900
mockito	MIT	25f13951d3	1791	3383
okhttp	Apache-2.0	2ece5518a2	74	29
pdfbox	Apache-2.0	0c4b77bc7d	1418	6930
pmd	BSD-4-Clause	2d7ff1a727	3475	12,765
retrofit	Apache-2.0	ee21a7aff2	510	1363
tika	Apache-2.0	d49b6d1d39	2174	7230

The comparatively sparse graph of okhttp (74 classes, 29 edges) reflects the fact that the core of that system is implemented in Kotlin, which is outside the scope of the Java-only extraction pipeline; only its Java sources contribute to the graph.

Table 2 provides a data dictionary for benchmark_summary.csv; the machine-readable data_dictionary.csv included in the dataset extends it to every file and attribute.

**Table 2:** Data dictionary for benchmark_summary.csv.

Column	Type	Description
project_name	string	Short identifier of the analyzed system
repository_url	string	Public GitHub URL of the source repository
license	string	SPDX license identifier of the system
commit_hash	string	Full SHA-1 of the analyzed commit
release_tag	string	Upstream release tag when the commit corresponds to a tagged release (optional; empty otherwise)
extraction_date	date	Date of graph extraction (ISO 8601)
jastg_version	string	JASTG version used for extraction
total_java_files	integer	Number of .java source files found under the project tree
parse_errors	integer	Number of .java files that failed to parse and were excluded
number_of_classes	integer	Number of nodes (classes and interfaces, including inner classes)
number_of_edges	integer	Number of directed dependency edges
graph_density	float	Density of the directed graph
average_degree	float	Mean total degree over all nodes
maximum_degree	integer	Highest total degree among all nodes

Fig. 1 presents the distribution of project sizes in terms of number of classes and number of edges. Values are displayed on a logarithmic scale. The dataset spans from small systems such as jpetstore-6 (43 classes) to large systems such as camel (29,695 classes).

**Fig. 1:**
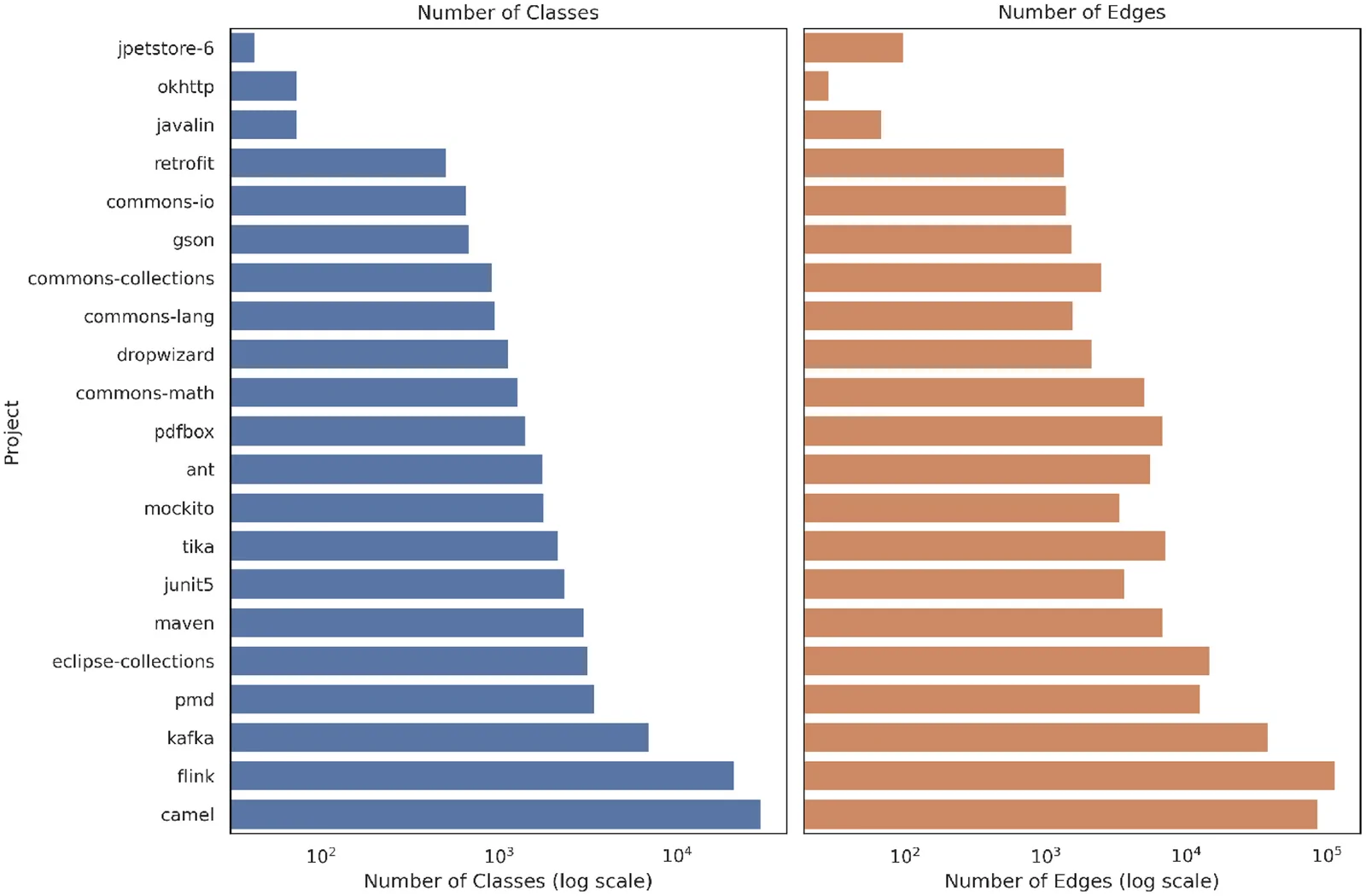
Distribution of project sizes in the dataset, measured by number of classes and number of edges. Values are shown on a logarithmic scale to account for differences in magnitude across systems. Source data: benchmark_summary.csv.

Fig. 2 provides a visual example of a structural dependency subgraph extracted from one of the analyzed systems. The visualization shows classes as nodes and typed dependencies as directed weighted edges. Test classes (e.g., CatalogServiceTest) appear as regular nodes because test sources are part of the analyzed source tree (see Experimental Design).

**Fig. 2:**
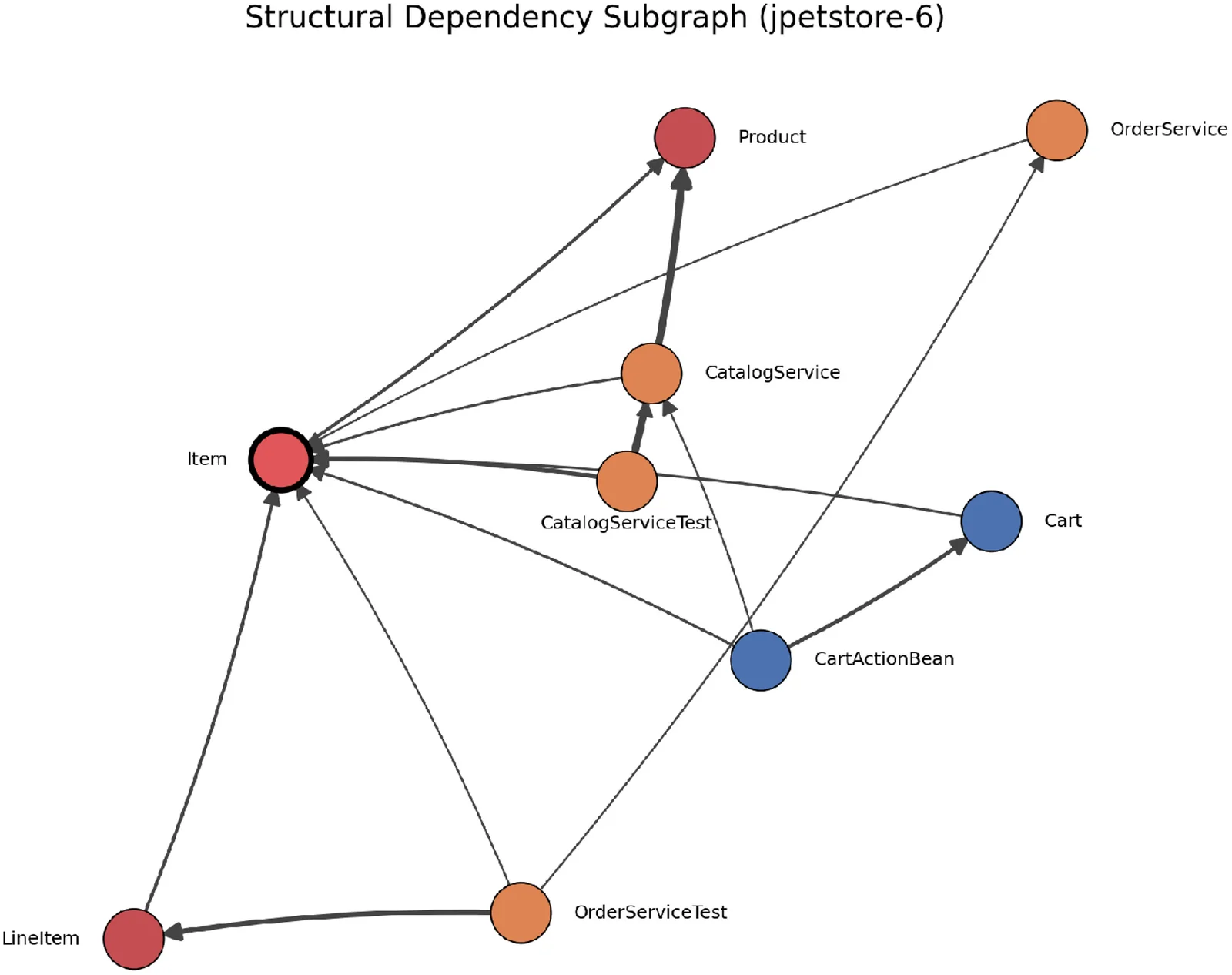
Example of a structural dependency subgraph extracted from the jpetstore-6 system, centered on the highly connected Item class. Nodes represent classes (labels simplified to class names), directed edges represent typed dependencies, and edge thickness is proportional to dependency weight. Source data: projects/jpetstore-6/graph_jpetstore-6.graphml.

Fig. 3 presents the distribution of selected object-oriented metrics across all classes in the dataset.

**Fig. 3:**
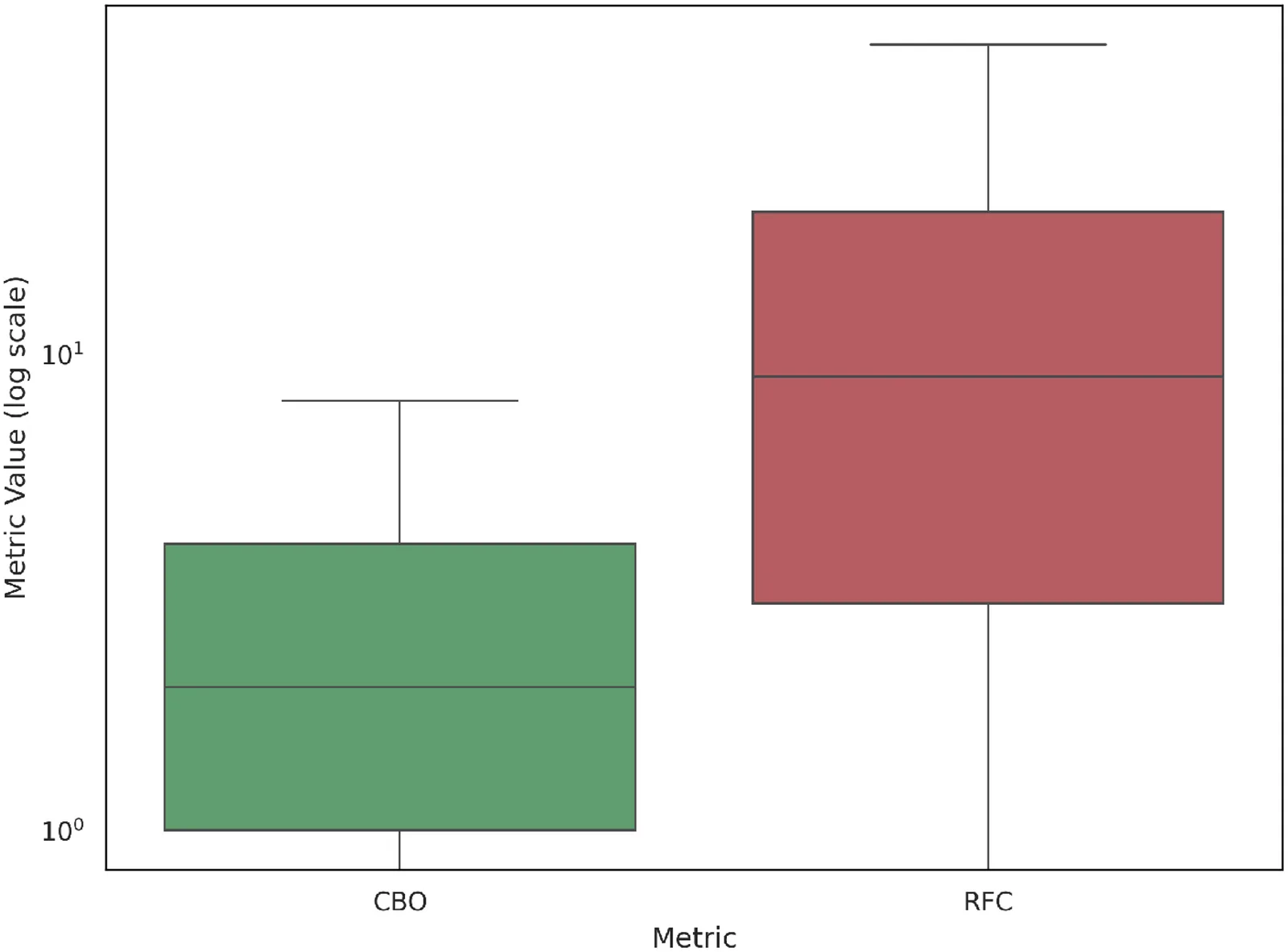
Distribution of object-oriented metrics across all classes in the dataset. Boxplots represent the variability of CBO and RFC values at the node level, displayed on a logarithmic scale. Source data: node attributes of all graph_<project>.graphml files.

Fig. 4, Fig. 5 present the per-project distribution of CBO and RFC values across the dataset.

**Fig. 4:**
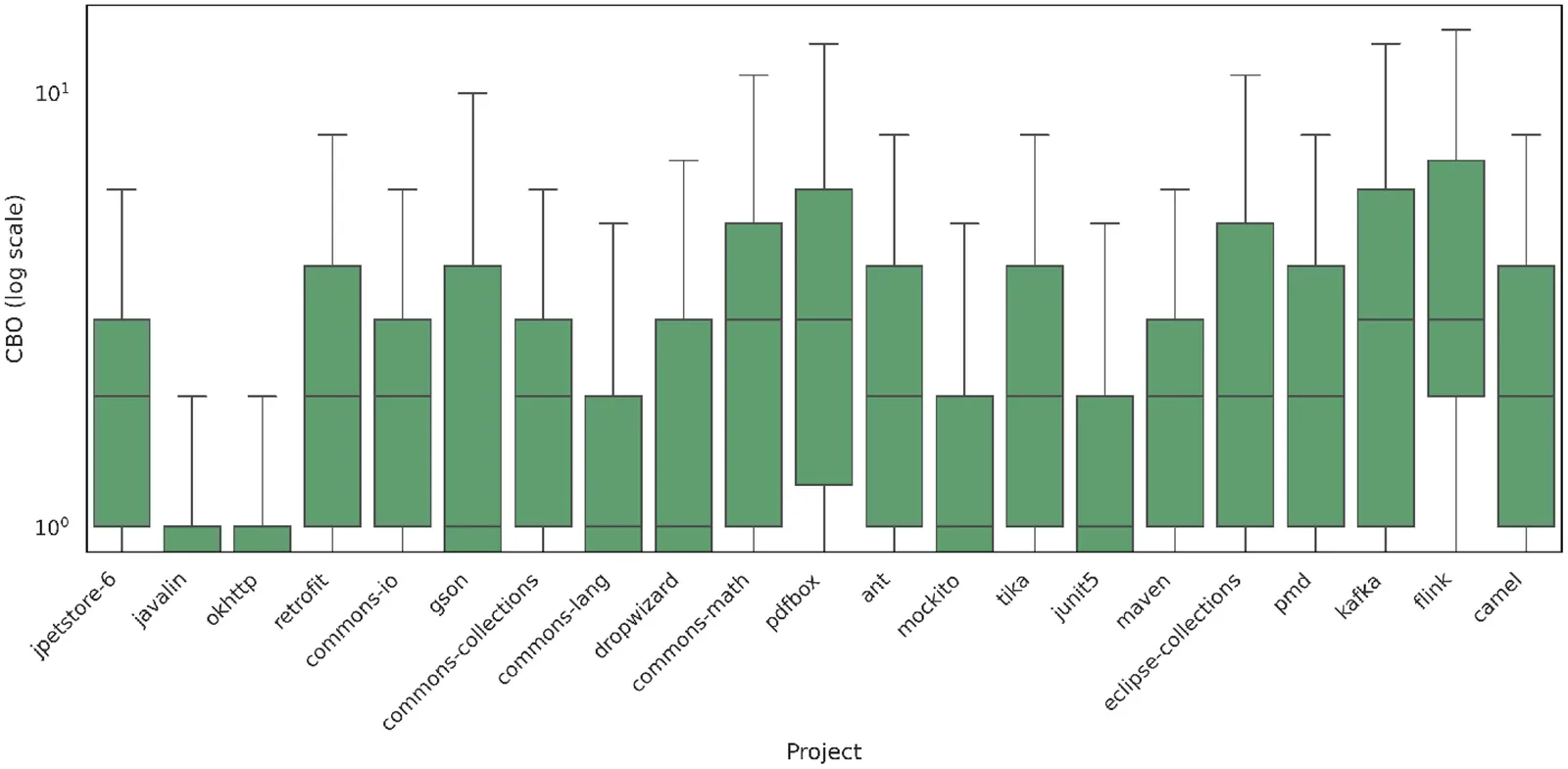
Distribution of CBO values across classes for each project. Boxplots represent variability within each system, displayed on a logarithmic scale. Source data: node attributes of all graph_<project>.graphml files.

**Fig. 5:**
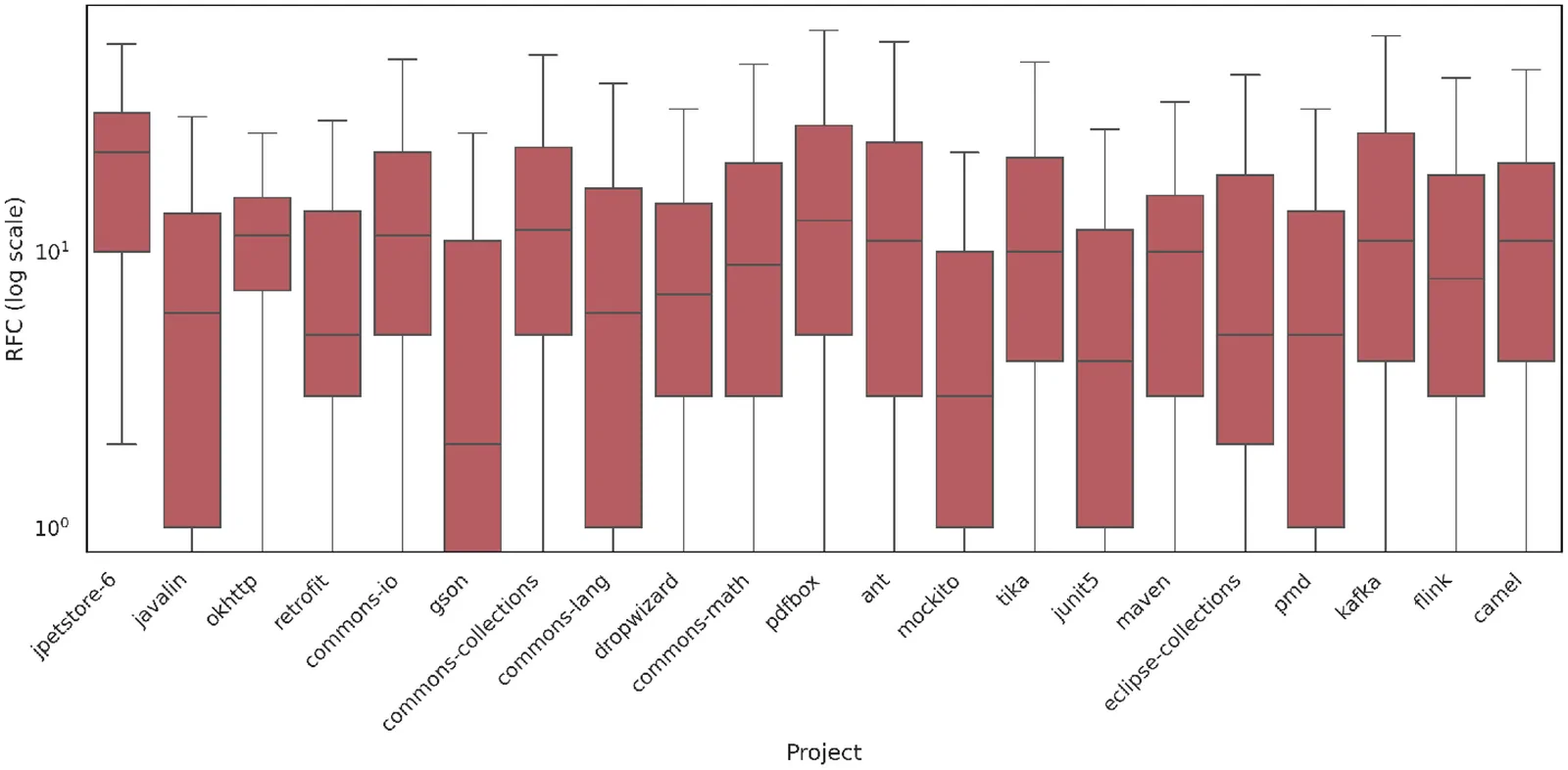
Distribution of RFC values across classes for each project. Boxplots represent variability within each system, displayed on a logarithmic scale. Source data: node attributes of all graph_<project>.graphml files.

Correspondence between the article and the dataset files: Fig. 1 was generated from benchmark_summary.csv (columns number_of_classes and number_of_edges); Fig. 2 was rendered from projects/jpetstore-6/graph_jpetstore-6.graphml; Fig. 3, Fig. 4, Fig. 5 were computed from the node-level CBO and RFC attributes of all graph_<project>.graphml files; and Table 1 corresponds to the columns project_name, license, commit_hash, number_of_classes, and number_of_edges of benchmark_summary.csv.

## 4 Experimental Design, Materials and Methods

Each repository was cloned and checked out at a fixed commit hash. Java source files were parsed into Abstract Syntax Tree representations using JASTG v1.0.0 (commit b62a943) [10]. Explicit typed dependencies were extracted between classes, including inheritance relationships, interface implementations, field type references, method parameter types, return types, and class instantiations. Dependency multiplicity was encoded as edge weights, while node identifiers are integers assigned deterministically by lexicographic sorting, with the fully qualified class name of each node stored in its label attribute. Graph-level statistics were computed and exported together with node-level metrics and execution metadata to ensure traceability.

**Extraction parameters and scope.** All 21 systems were analyzed with the default JASTG configuration: weighted directed graphs and the upper qualifier heuristic for resolving static method invocation qualifiers [2]. The tool scans all .java files under the project tree without requiring compilation or classpath access; consequently, test sources are included in the analysis and appear as regular nodes, and references to external library classes are not represented (the CBO and RFC attributes are therefore lower bounds with respect to their canonical definitions, as discussed in the companion tool paper [2]). Source files written in other JVM languages (e.g., Kotlin) are not analyzed. Files that cannot be parsed by the underlying javalang parser — typically because they use Java language constructs introduced after the grammar supported by javalang 0.13.0, such as records, sealed classes, and pattern matching — are skipped and counted in the parse_errors field of each project's metadata. Across the corpus, parse failures affected 2464 of 65,384 source files (3.8%), with a median per-system rate of 1.7%; the highest rates occur in okhttp (21.1%, a Kotlin-core project whose Java sources are peripheral) and junit5 (17.5%, a codebase with extensive adoption of modern Java features). Per-system counts are recorded in the total_java_files and parse_errors columns of benchmark_summary.csv and in each metadata file, making the exclusion rate auditable per project. A detailed characterization of parse coverage and extraction performance (wall-clock time and peak memory per system) is provided in the companion tool paper [2].

**Execution environment and reproducibility.** The archived dataset was extracted on Arch Linux (kernel 6.18.9, x86_64) using Python 3.14.3, javalang 0.13.0, and networkx 3.6.1, as recorded in each metadata file. JASTG produces deterministic output: file traversal and node identifier assignment follow lexicographic order, so repeated runs on the same source tree yield identical graphs. Each metadata file additionally records a config_hash — the SHA-256 digest of the analysis parameters (domain names and paths, graph mode, and qualifier heuristic) — which, combined with the recorded tool and dependency versions and the source commit hash, constitutes a verifiable reproducibility fingerprint: a reproduction run with the same parameters yields an identical hash.

**Worked example.** The following steps regenerate the graph and metadata for the smallest system in the dataset (jpetstore-6); JASTG is distributed on PyPI and requires Python 3.10 or later:

**Table:** 

pip install jastg

mkdir -p projects
git clonehttps://github.com/mybatis/jpetstore-6projects/jpetstore-6
git -C projects/jpetstore-6 checkout 41207bd96c35dce3e746d4af0fdf343f4560ea68

jastg analyze –domain jpetstore-6 –path projects/jpetstore-6
This produces graph_jpetstore-6. graphml and metadata_jpetstore-6. json in output/jpetstore-6/, matching the archived artifacts, including the configuration hash shown above. The same command pattern applies to all 21 systems, whose repository URLs and commit hashes are listed in projects.csv.

The dataset is permanently archived on Zenodo under the concept DOI 10.5281/zenodo.18744312 [13], which always resolves to the latest version. The current release, v1.0.1 (version DOI: 10.5281/zenodo.21221557), extends the initial release v1.0.0 (version DOI: 10.5281/zenodo.18744313) with the total_java_files and parse_errors columns in benchmark_summary.csv, the machine-readable data_dictionary.csv, and an expanded README with loading examples; all GraphML and JSON artifacts are identical to v1.0.0 and immutable under their DOI versions. The source code of JASTG is publicly available on GitHub at https://github.com/MarcosCordeiro/jastg, and archived on Zenodo [10], enabling independent regeneration of the dataset from the original repositories and commits.

## Limitations

The dataset captures only explicit typed structural dependencies derived from source-level AST analysis. It does not include dynamic runtime dependencies, reflection-based interactions, or full semantic type resolution. In addition, source files using Java language constructs outside the grammar supported by the underlying parser are excluded from the graphs, affecting 3.8% of source files across the corpus (see Experimental Design). Furthermore, each project is represented by a single fixed commit and therefore does not reflect longitudinal evolution across versions.

These limitations have practical implications for dataset selection. Because dynamic and reflection-based dependencies are absent, the graphs may underestimate coupling in systems that rely heavily on dependency injection frameworks or reflective mechanisms; the dataset is therefore best suited for studies of static structural organization, modularity, and design metrics, rather than runtime architecture analysis or dynamic dependency mining. Graphs of systems with high parse-failure rates or with substantial non-Java code (most notably junit5 and okhttp) under-represent the full system and should be interpreted accordingly; the exact exclusion rate of every system is auditable through the published metadata. Similarly, the single-commit design supports controlled cross-system comparison but not software evolution studies; researchers interested in longitudinal analyses can nonetheless regenerate comparable graphs for additional commits using the publicly available JASTG pipeline.

## Ethics Statement

The authors have read and follow the ethical requirements for publication in Data in Brief and confirm that the current work does not involve human subjects, animal experiments, or any data collected from social media platforms.

## Credit Author Statement

**Marcos Cordeiro de Brito Jr.:** Conceptualization, Methodology, Software, Data curation, Writing – original draft, Visualization.

**Leandro Augusto da Silva:** Supervision, Validation, Writing – review & editing.

## Declaration of Generative AI and AI-Assisted Technologies in the Manuscript Preparation Process

During the preparation of this work the authors used ChatGPT and Claude in order to improve language and readability. After using these tools/services, the authors reviewed and edited the content as needed and take full responsibility for the content of the published article.

## Data Availability

ZenodoJASTG Structural Graph Benchmark (Original data).
